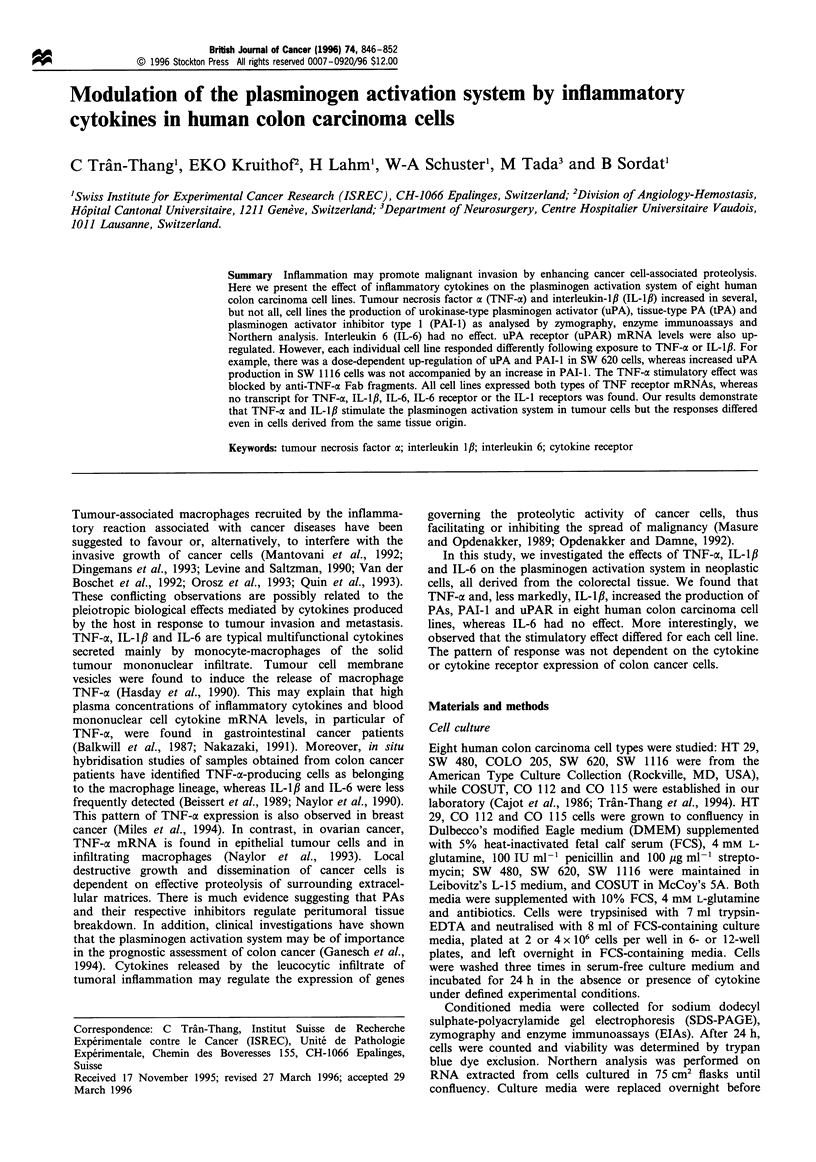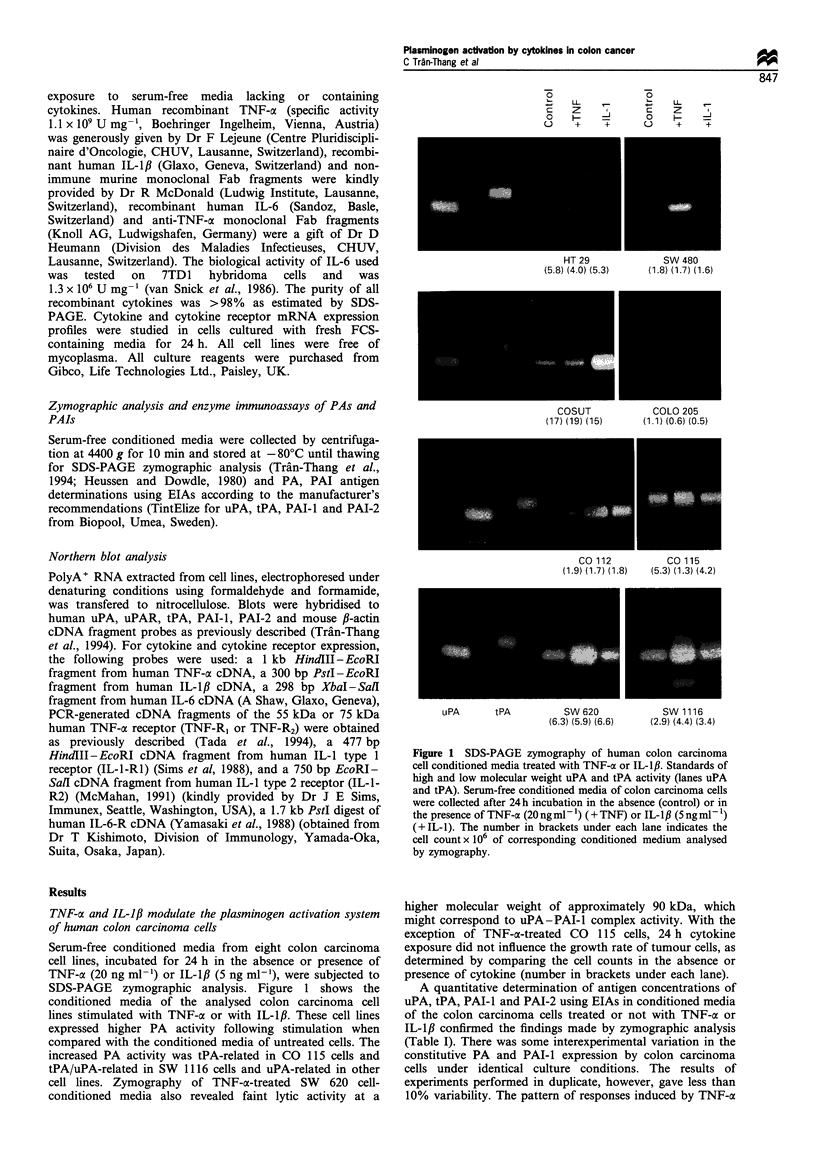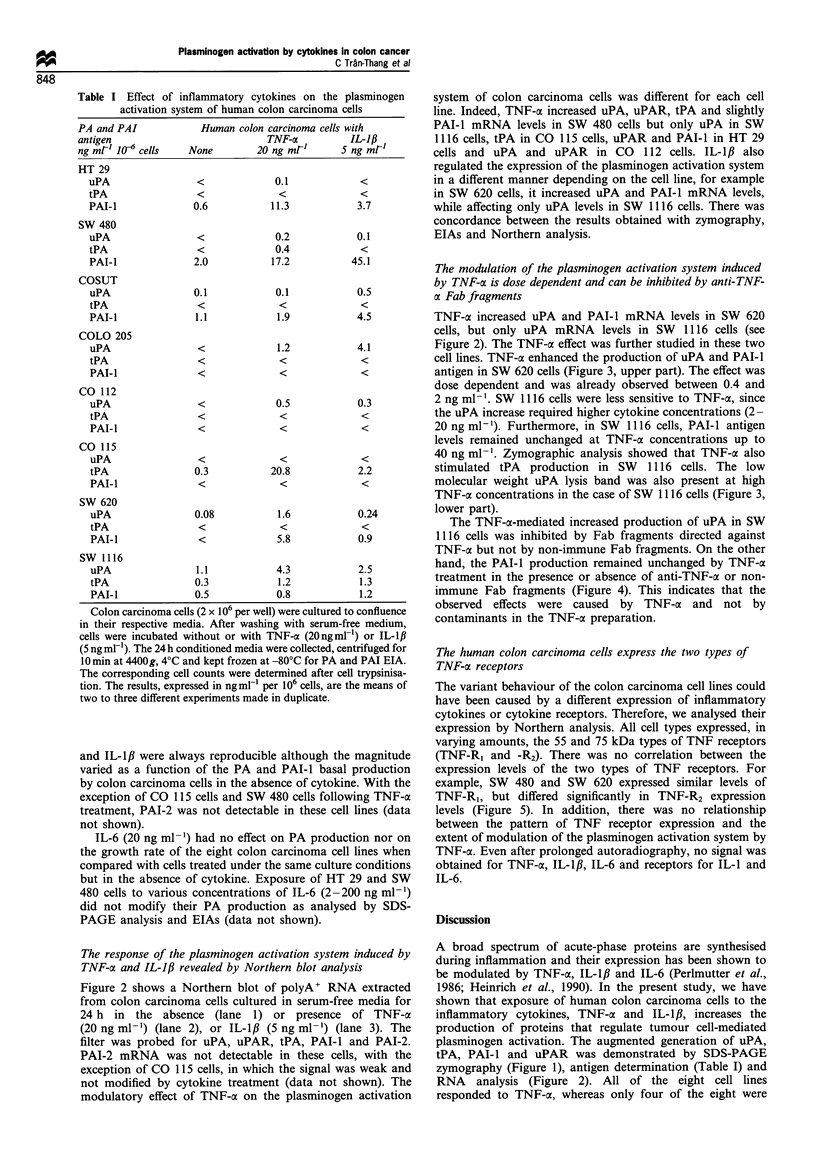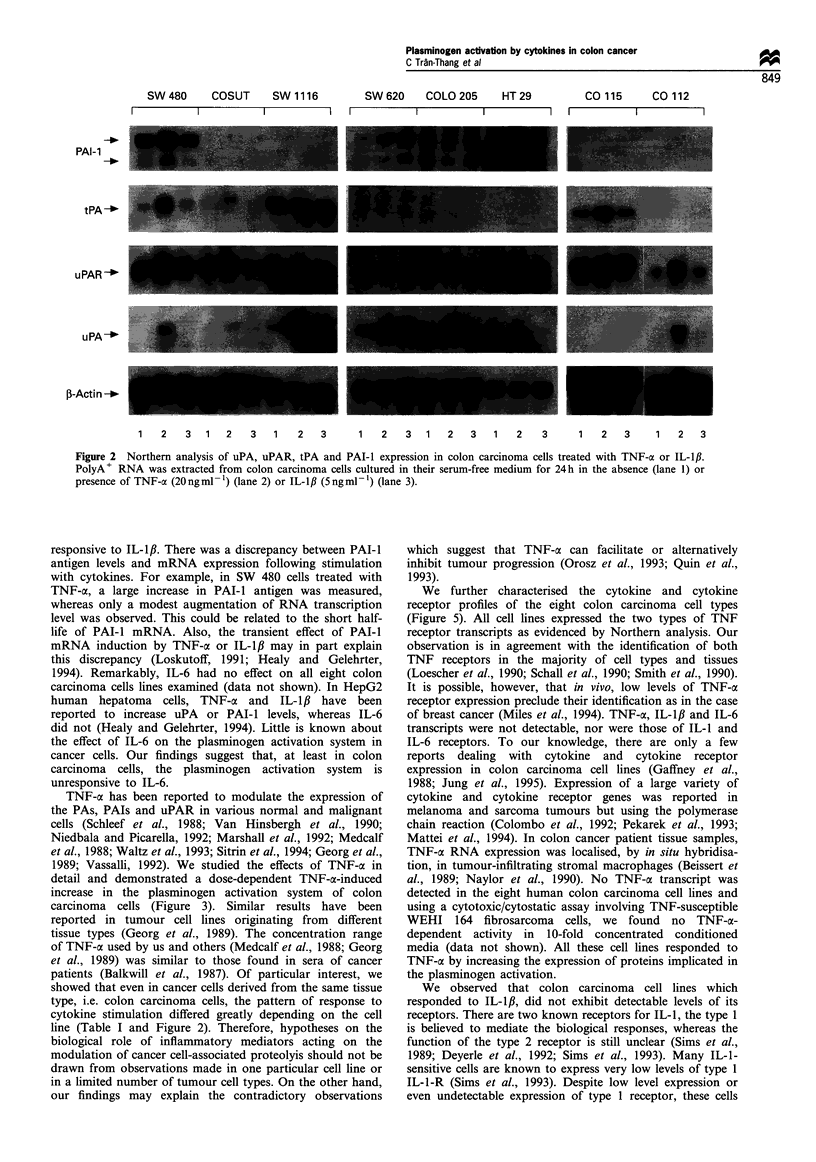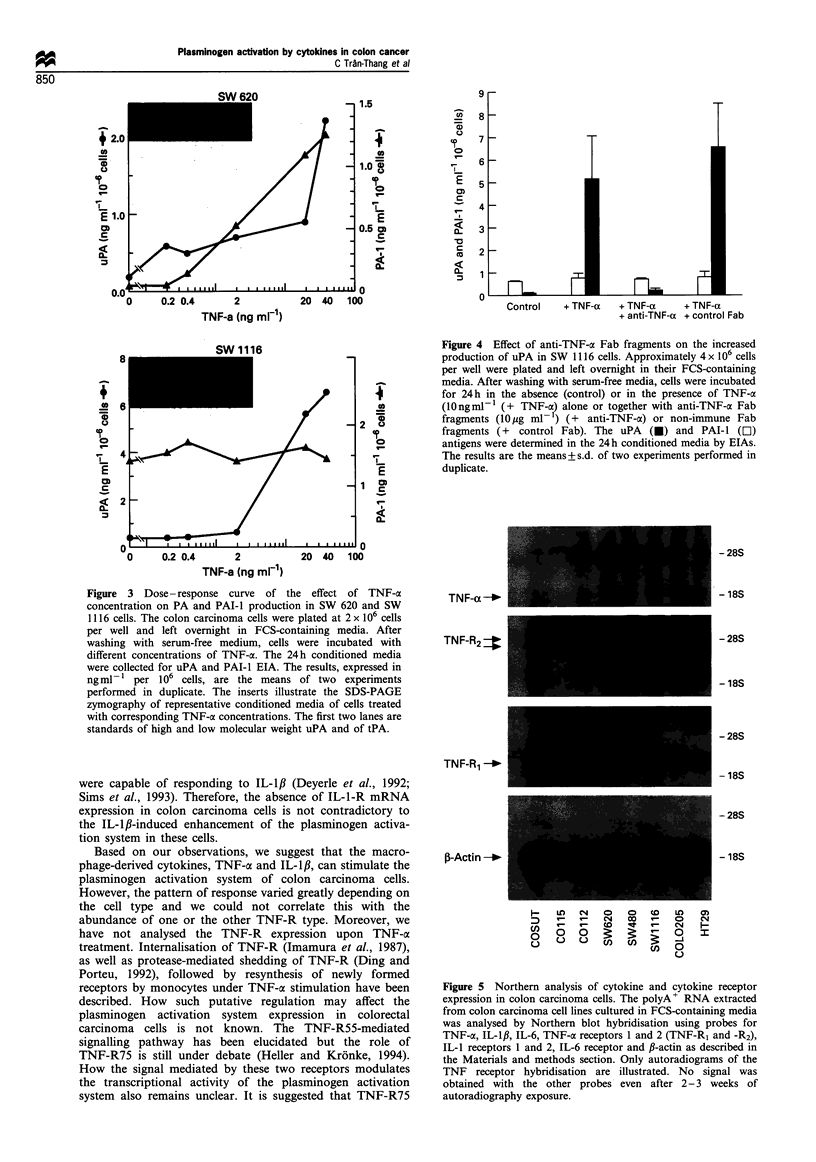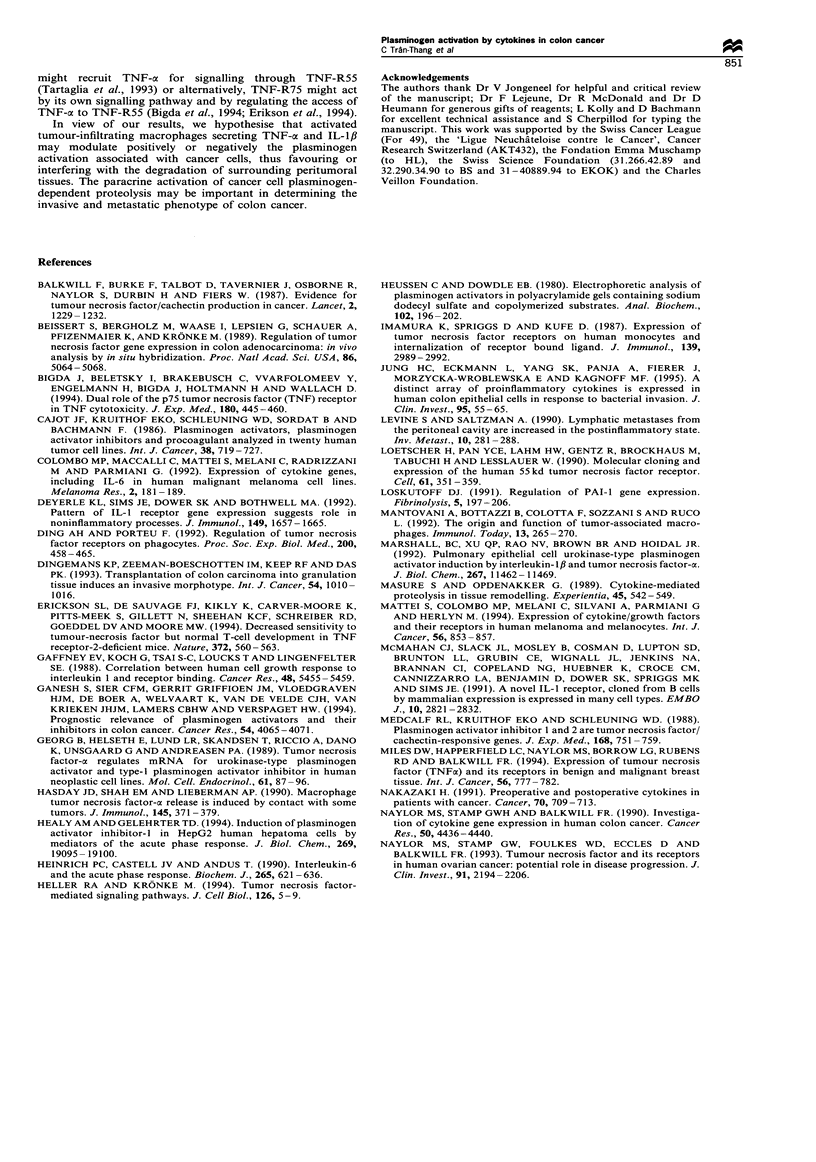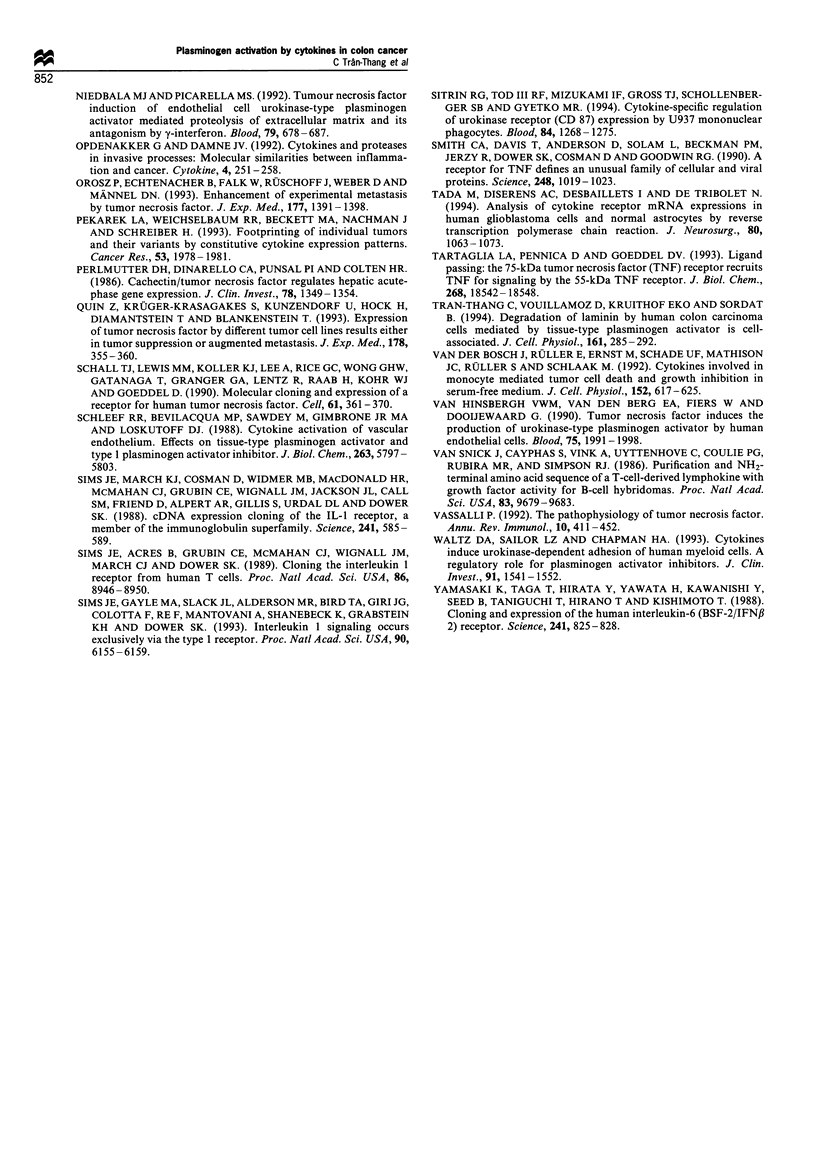# Modulation of the plasminogen activation system by inflammatory cytokines in human colon carcinoma cells.

**DOI:** 10.1038/bjc.1996.447

**Published:** 1996-09

**Authors:** C. Trân-Thang, E. Kruithof, H. Lahm, W. A. Schuster, M. Tada, B. Sordat

**Affiliations:** Swiss Institute for Experimental Cancer Research (ISREC), Epalinges, Switzerland.

## Abstract

**Images:**


British Journal of Cancer (1996) 74, 846-852
© 1996 Stockton Press All rights reserved 0007-0920/96 $12.00

### Modulation of the plasminogen activation system by inflammatory cytokines in human colon carcinoma cells

C Trân-Thang[1], EKO Kruithof[2], H Lahm[1], W-A Schuster[1], M Tada[3] and B Sordat[1]

[1]*Swiss Institute for Experimental Cancer Research (ISREC), CH-1066 Epalinges, Switzerland;* [2]*Division of Angiology-Hemostasis, Hôpital Cantonal Universitaire, 1211 Genève, Switzerland;* [3]*Department of Neurosurgery, Centre Hospitalier Universitaire Vaudois, 1011 Lausanne, Switzerland.*

**Summary** Inflammation may promote malignant invasion by enhancing cancer cell-associated proteolysis. Here we present the effect of inflammatory cytokines on the plasminogen activation system of eight human colon carcinoma cell lines. Tumour necrosis factor α (TNF-α) and interleukin-1β (IL-1β) increased in several, but not all, cell lines the production of urokinase-type plasminogen activator (uPA), tissue-type PA (tPA) and plasminogen activator inhibitor type 1 (PAI-1) as analysed by zymography, enzyme immunoassays and Northern analysis. Interleukin 6 (IL-6) had no effect. uPA receptor (uPAR) mRNA levels were also up-regulated. However, each individual cell line responded differently following exposure to TNF-α or IL-1β. For example, there was a dose-dependent up-regulation of uPA and PAI-1 in SW 620 cells, whereas increased uPA production in SW 1116 cells was not accompanied by an increase in PAI-1. The TNF-α stimulatory effect was blocked by anti-TNF-α Fab fragments. All cell lines expressed both types of TNF receptor mRNAs, whereas no transcript for TNF-α, IL-1β, IL-6, IL-6 receptor or the IL-1 receptors was found. Our results demonstrate that TNF-α and IL-1β stimulate the plasminogen activation system in tumour cells but the responses differed even in cells derived from the same tissue origin.

**Keywords:** tumour necrosis factor α; interleukin 1β; interleukin 6; cytokine receptor

Tumour-associated macrophages recruited by the inflammatory reaction associated with cancer diseases have been suggested to favour or, alternatively, to interfere with the invasive growth of cancer cells (Mantovani *et al.*, 1992; Dingemans *et al.*, 1993; Levine and Saltzman, 1990; Van der Boschet *et al.*, 1992; Orosz *et al.*, 1993; Quin *et al.*, 1993). These conflicting observations are possibly related to the pleiotropic biological effects mediated by cytokines produced by the host in response to tumour invasion and metastasis. TNF-α, IL-1β and IL-6 are typical multifunctional cytokines secreted mainly by monocyte-macrophages of the solid tumour mononuclear infiltrate. Tumour cell membrane vesicles were found to induce the release of macrophage TNF-α (Hasday *et al.*, 1990). This may explain that high plasma concentrations of inflammatory cytokines and blood mononuclear cell cytokine mRNA levels, in particular of TNF-α, were found in gastrointestinal cancer patients (Balkwill *et al.*, 1987; Nakazaki, 1991). Moreover, *in situ* hybridisation studies of samples obtained from colon cancer patients have identified TNF-α-producing cells as belonging to the macrophage lineage, whereas IL-1β and IL-6 were less frequently detected (Beissert *et al.*, 1989; Naylor *et al.*, 1990). This pattern of TNF-α expression is also observed in breast cancer (Miles *et al.*, 1994). In contrast, in ovarian cancer, TNF-α mRNA is found in epithelial tumour cells and in infiltrating macrophages (Naylor *et al.*, 1993). Local destructive growth and dissemination of cancer cells is dependent on effective proteolysis of surrounding extracellular matrices. There is much evidence suggesting that PAs and their respective inhibitors regulate peritumoral tissue breakdown. In addition, clinical investigations have shown that the plasminogen activation system may be of importance in the prognostic assessment of colon cancer (Ganesch *et al.*, 1994). Cytokines released by the leucocytic infiltrate of tumoral inflammation may regulate the expression of genes governing the proteolytic activity of cancer cells, thus facilitating or inhibiting the spread of malignancy (Masure and Opdenakker, 1989; Opdenakker and Damne, 1992).

In this study, we investigated the effects of TNF-α, IL-1β and IL-6 on the plasminogen activation system in neoplastic cells, all derived from the colorectal tissue. We found that TNF-α and, less markedly, IL-1β, increased the production of PAs, PAI-1 and uPAR in eight human colon carcinoma cell lines, whereas IL-6 had no effect. More interestingly, we observed that the stimulatory effect differed for each cell line. The pattern of response was not dependent on the cytokine or cytokine receptor expression of colon cancer cells.

Correspondence: C Trân-Thang, Institut Suisse de Recherche Expérimentale contre le Cancer (ISREC), Unité de Pathologie Expérimentale, Chemin des Boveresses 155, CH-1066 Epalinges, Suisse
Received 17 November 1995; revised 27 March 1996; accepted 29 March 1996

### Materials and methods

### *Cell culture*

Eight human colon carcinoma cell types were studied: HT 29, SW 480, COLO 205, SW 620, SW 1116 were from the American Type Culture Collection (Rockville, MD, USA), while COSUT, CO 112 and CO 115 were established in our laboratory (Cajot *et al.*, 1986; Trân-Thang *et al.*, 1994). HT 29, CO 112 and CO 115 cells were grown to confluency in Dulbecco's modified Eagle medium (DMEM) supplemented with 5% heat-inactivated fetal calf serum (FCS), 4 mM L-glutamine, 100 IU $ml^{-1}$ penicillin and 100 $\mu g\ ml^{-1}$ streptomycin; SW 480, SW 620, SW 1116 were maintained in Leibovitz's L-15 medium, and COSUT in McCoy's 5A. Both media were supplemented with 10% FCS, 4 mM L-glutamine and antibiotics. Cells were trypsinised with 7 ml trypsin-EDTA and neutralised with 8 ml of FCS-containing culture media, plated at 2 or $4 \times 10^6$ cells per well in 6- or 12-well plates, and left overnight in FCS-containing media. Cells were washed three times in serum-free culture medium and incubated for 24 h in the absence or presence of cytokine under defined experimental conditions.

Conditioned media were collected for sodium dodecyl sulphate-polyacrylamide gel electrophoresis (SDS-PAGE), zymography and enzyme immunoassays (EIAs). After 24 h, cells were counted and viability was determined by trypan blue dye exclusion. Northern analysis was performed on RNA extracted from cells cultured in 75 $cm^2$ flasks until confluency. Culture media were replaced overnight before exposure to serum-free media lacking or containing cytokines. Human recombinant TNF-α (specific activity $1.1 \times 10^9$ U $mg^{-1}$, Boehringer Ingelheim, Vienna, Austria) was generously given by Dr F Lejeune (Centre Pluridisciplinaire d'Oncologie, CHUV, Lausanne, Switzerland), recombinant human IL-1β (Glaxo, Geneva, Switzerland) and non-immune murine monoclonal Fab fragments were kindly provided by Dr R McDonald (Ludwig Institute, Lausanne, Switzerland), recombinant human IL-6 (Sandoz, Basle, Switzerland) and anti-TNF-α monoclonal Fab fragments (Knoll AG, Ludwigshafen, Germany) were a gift of Dr D Heumann (Division des Maladies Infectieuses, CHUV, Lausanne, Switzerland). The biological activity of IL-6 used was tested on 7TD1 hybridoma cells and was $1.3 \times 10^6$ U $mg^{-1}$ (van Snick *et al.*, 1986). The purity of all recombinant cytokines was >98% as estimated by SDS-PAGE. Cytokine and cytokine receptor mRNA expression profiles were studied in cells cultured with fresh FCS-containing media for 24 h. All cell lines were free of mycoplasma. All culture reagents were purchased from Gibco, Life Technologies Ltd., Paisley, UK.

*Zymographic analysis and enzyme immunoassays of PAs and PAIs*

Serum-free conditioned media were collected by centrifugation at 4400 ***g*** for 10 min and stored at −80°C until thawing for SDS-PAGE zymographic analysis (Trân-Thang *et al.*, 1994; Heussen and Dowdle, 1980) and PA, PAI antigen determinations using EIAs according to the manufacturer's recommendations (TintElize for uPA, tPA, PAI-1 and PAI-2 from Biopool, Umea, Sweden).

*Northern blot analysis*

PolyA$^+$ RNA extracted from cell lines, electrophoresed under denaturing conditions using formaldehyde and formamide, was transfered to nitrocellulose. Blots were hybridised to human uPA, uPAR, tPA, PAI-1, PAI-2 and mouse β-actin cDNA fragment probes as previously described (Trân-Thang *et al.*, 1994). For cytokine and cytokine receptor expression, the following probes were used: a 1 kb *Hin*dIII−*Eco*RI fragment from human TNF-α cDNA, a 300 bp *Pst*I−*Eco*RI fragment from human IL-1β cDNA, a 298 bp *Xba*I−*Sal*I fragment from human IL-6 cDNA (A Shaw, Glaxo, Geneva), PCR-generated cDNA fragments of the 55 kDa or 75 kDa human TNF-α receptor (TNF-$R_1$ or TNF-$R_2$) were obtained as previously described (Tada *et al.*, 1994), a 477 bp *Hin*dIII−*Eco*RI cDNA fragment from human IL-1 type 1 receptor (IL-1-R1) (Sims *et al*, 1988), and a 750 bp *Eco*RI−*Sal*I cDNA fragment from human IL-1 type 2 receptor (IL-1-R2) (McMahan, 1991) (kindly provided by Dr J E Sims, Immunex, Seattle, Washington, USA), a 1.7 kb *Pst*I digest of human IL-6-R cDNA (Yamasaki *et al.*, 1988) (obtained from Dr T Kishimoto, Division of Immunology, Yamada-Oka, Suita, Osaka, Japan).

**Figure 1** SDS-PAGE zymography of human colon carcinoma cell conditioned media treated with TNF-α or IL-1β. Standards of high and low molecular weight uPA and tPA activity (lanes uPA and tPA). Serum-free conditioned media of colon carcinoma cells were collected after 24 h incubation in the absence (control) or in the presence of TNF-α (20 ng $ml^{-1}$) (+TNF) or IL-1β (5 ng $ml^{-1}$) (+IL-1). The number in brackets under each lane indicates the cell count $\times 10^6$ of corresponding conditioned medium analysed by zymography.

### Results

*TNF-α and IL-1β modulate the plasminogen activation system of human colon carcinoma cells*

Serum-free conditioned media from eight colon carcinoma cell lines, incubated for 24 h in the absence or presence of TNF-α (20 ng $ml^{-1}$) or IL-1β (5 ng $ml^{-1}$), were subjected to SDS-PAGE zymographic analysis. Figure 1 shows the conditioned media of the analysed colon carcinoma cell lines stimulated with TNF-α or with IL-1β. These cell lines expressed higher PA activity following stimulation when compared with the conditioned media of untreated cells. The increased PA activity was tPA-related in CO 115 cells and tPA/uPA-related in SW 1116 cells and uPA-related in other cell lines. Zymography of TNF-α-treated SW 620 cell-conditioned media also revealed faint lytic activity at a higher molecular weight of approximately 90 kDa, which might correspond to uPA−PAI-1 complex activity. With the exception of TNF-α-treated CO 115 cells, 24 h cytokine exposure did not influence the growth rate of tumour cells, as determined by comparing the cell counts in the absence or presence of cytokine (number in brackets under each lane).

A quantitative determination of antigen concentrations of uPA, tPA, PAI-1 and PAI-2 using EIAs in conditioned media of the colon carcinoma cells treated or not with TNF-α or IL-1β confirmed the findings made by zymographic analysis (Table I). There was some interexperimental variation in the constitutive PA and PAI-1 expression by colon carcinoma cells under identical culture conditions. The results of experiments performed in duplicate, however, gave less than 10% variability. The pattern of responses induced by TNF-α

**Table I** Effect of inflammatory cytokines on the plasminogen activation system of human colon carcinoma cells

| *PA and PAI antigen ng ml$^{-1}$ 10$^{-6}$ cells* | *Human colon carcinoma cells with* None | *TNF-α 20 ng ml$^{-1}$* | *IL-1β 5 ng ml$^{-1}$* |
|---|---|---|---|
| HT 29 | | | |
| uPA | < | 0.1 | < |
| tPA | < | < | < |
| PAI-1 | 0.6 | 11.3 | 3.7 |
| SW 480 | | | |
| uPA | < | 0.2 | 0.1 |
| tPA | < | 0.4 | < |
| PAI-1 | 2.0 | 17.2 | 45.1 |
| COSUT | | | |
| uPA | 0.1 | 0.1 | 0.5 |
| tPA | < | < | < |
| PAI-1 | 1.1 | 1.9 | 4.5 |
| COLO 205 | | | |
| uPA | < | 1.2 | 4.1 |
| tPA | < | < | < |
| PAI-1 | < | < | < |
| CO 112 | | | |
| uPA | < | 0.5 | 0.3 |
| tPA | < | < | < |
| PAI-1 | < | < | < |
| CO 115 | | | |
| uPA | < | < | < |
| tPA | 0.3 | 20.8 | 2.2 |
| PAI-1 | < | < | < |
| SW 620 | | | |
| uPA | 0.08 | 1.6 | 0.24 |
| tPA | < | < | < |
| PAI-1 | < | 5.8 | 0.9 |
| SW 1116 | | | |
| uPA | 1.1 | 4.3 | 2.5 |
| tPA | 0.3 | 1.2 | 1.3 |
| PAI-1 | 0.5 | 0.8 | 1.2 |

Colon carcinoma cells ($2 \times 10^6$ per well) were cultured to confluence in their respective media. After washing with serum-free medium, cells were incubated without or with TNF-α (20 ng ml$^{-1}$) or IL-1β (5 ng ml$^{-1}$). The 24 h conditioned media were collected, centrifuged for 10 min at 4400 *g*, 4°C and kept frozen at –80°C for PA and PAI EIA. The corresponding cell counts were determined after cell trypsinisation. The results, expressed in ng ml$^{-1}$ per $10^6$ cells, are the means of two to three different experiments made in duplicate.

and IL-1β were always reproducible although the magnitude varied as a function of the PA and PAI-1 basal production by colon carcinoma cells in the absence of cytokine. With the exception of CO 115 cells and SW 480 cells following TNF-α treatment, PAI-2 was not detectable in these cell lines (data not shown).

IL-6 (20 ng ml$^{-1}$) had no effect on PA production nor on the growth rate of the eight colon carcinoma cell lines when compared with cells treated under the same culture conditions but in the absence of cytokine. Exposure of HT 29 and SW 480 cells to various concentrations of IL-6 (2–200 ng ml$^{-1}$) did not modify their PA production as analysed by SDS-PAGE analysis and EIAs (data not shown).

*The response of the plasminogen activation system induced by TNF-α and IL-1β revealed by Northern blot analysis*

Figure 2 shows a Northern blot of polyA$^+$ RNA extracted from colon carcinoma cells cultured in serum-free media for 24 h in the absence (lane 1) or presence of TNF-α (20 ng ml$^{-1}$) (lane 2), or IL-1β (5 ng ml$^{-1}$) (lane 3). The filter was probed for uPA, uPAR, tPA, PAI-1 and PAI-2. PAI-2 mRNA was not detectable in these cells, with the exception of CO 115 cells, in which the signal was weak and not modified by cytokine treatment (data not shown). The modulatory effect of TNF-α on the plasminogen activation system of colon carcinoma cells was different for each cell line. Indeed, TNF-α increased uPA, uPAR, tPA and slightly PAI-1 mRNA levels in SW 480 cells but only uPA in SW 1116 cells, tPA in CO 115 cells, uPAR and PAI-1 in HT 29 cells and uPA and uPAR in CO 112 cells. IL-1β also regulated the expression of the plasminogen activation system in a different manner depending on the cell line, for example in SW 620 cells, it increased uPA and PAI-1 mRNA levels, while affecting only uPA levels in SW 1116 cells. There was concordance between the results obtained with zymography, EIAs and Northern analysis.

*The modulation of the plasminogen activation system induced by TNF-α is dose dependent and can be inhibited by anti-TNF-α Fab fragments*

TNF-α increased uPA and PAI-1 mRNA levels in SW 620 cells, but only uPA mRNA levels in SW 1116 cells (see Figure 2). The TNF-α effect was further studied in these two cell lines. TNF-α enhanced the production of uPA and PAI-1 antigen in SW 620 cells (Figure 3, upper part). The effect was dose dependent and was already observed between 0.4 and 2 ng ml$^{-1}$. SW 1116 cells were less sensitive to TNF-α, since the uPA increase required higher cytokine concentrations (2–20 ng ml$^{-1}$). Furthermore, in SW 1116 cells, PAI-1 antigen levels remained unchanged at TNF-α concentrations up to 40 ng ml$^{-1}$. Zymographic analysis showed that TNF-α also stimulated tPA production in SW 1116 cells. The low molecular weight uPA lysis band was also present at high TNF-α concentrations in the case of SW 1116 cells (Figure 3, lower part).

The TNF-α-mediated increased production of uPA in SW 1116 cells was inhibited by Fab fragments directed against TNF-α but not by non-immune Fab fragments. On the other hand, the PAI-1 production remained unchanged by TNF-α treatment in the presence or absence of anti-TNF-α or non-immune Fab fragments (Figure 4). This indicates that the observed effects were caused by TNF-α and not by contaminants in the TNF-α preparation.

*The human colon carcinoma cells express the two types of TNF-α receptors*

The variant behaviour of the colon carcinoma cell lines could have been caused by a different expression of inflammatory cytokines or cytokine receptors. Therefore, we analysed their expression by Northern analysis. All cell types expressed, in varying amounts, the 55 and 75 kDa types of TNF receptors (TNF-$R_1$ and -$R_2$). There was no correlation between the expression levels of the two types of TNF receptors. For example, SW 480 and SW 620 expressed similar levels of TNF-$R_1$, but differed significantly in TNF-$R_2$ expression levels (Figure 5). In addition, there was no relationship between the pattern of TNF receptor expression and the extent of modulation of the plasminogen activation system by TNF-α. Even after prolonged autoradiography, no signal was obtained for TNF-α, IL-1β, IL-6 and receptors for IL-1 and IL-6.

### Discussion

A broad spectrum of acute-phase proteins are synthesised during inflammation and their expression has been shown to be modulated by TNF-α, IL-1β and IL-6 (Perlmutter *et al.*, 1986; Heinrich *et al.*, 1990). In the present study, we have shown that exposure of human colon carcinoma cells to the inflammatory cytokines, TNF-α and IL-1β, increases the production of proteins that regulate tumour cell-mediated plasminogen activation. The augmented generation of uPA, tPA, PAI-1 and uPAR was demonstrated by SDS-PAGE zymography (Figure 1), antigen determination (Table I) and RNA analysis (Figure 2). All of the eight cell lines responded to TNF-α, whereas only four of the eight were

**Figure 2** Northern analysis of uPA, uPAR, tPA and PAI-1 expression in colon carcinoma cells treated with TNF-α or IL-1β. PolyA$^+$ RNA was extracted from colon carcinoma cells cultured in their serum-free medium for 24 h in the absence (lane 1) or presence of TNF-α (20 ng ml$^{-1}$) (lane 2) or IL-1β (5 ng ml$^{-1}$) (lane 3).

responsive to IL-1β. There was a discrepancy between PAI-1 antigen levels and mRNA expression following stimulation with cytokines. For example, in SW 480 cells treated with TNF-α, a large increase in PAI-1 antigen was measured, whereas only a modest augmentation of RNA transcription level was observed. This could be related to the short half-life of PAI-1 mRNA. Also, the transient effect of PAI-1 mRNA induction by TNF-α or IL-1β may in part explain this discrepancy (Loskutoff, 1991; Healy and Gelehrter, 1994). Remarkably, IL-6 had no effect on all eight colon carcinoma cells lines examined (data not shown). In HepG2 human hepatoma cells, TNF-α and IL-1β have been reported to increase uPA or PAI-1 levels, whereas IL-6 did not (Healy and Gelehrter, 1994). Little is known about the effect of IL-6 on the plasminogen activation system in cancer cells. Our findings suggest that, at least in colon carcinoma cells, the plasminogen activation system is unresponsive to IL-6.

TNF-α has been reported to modulate the expression of the PAs, PAIs and uPAR in various normal and malignant cells (Schleef *et al.*, 1988; Van Hinsbergh *et al.*, 1990; Niedbala and Picarella, 1992; Marshall *et al.*, 1992; Medcalf *et al.*, 1988; Waltz *et al.*, 1993; Sitrin *et al.*, 1994; Georg *et al.*, 1989; Vassalli, 1992). We studied the effects of TNF-α in detail and demonstrated a dose-dependent TNF-α-induced increase in the plasminogen activation system of colon carcinoma cells (Figure 3). Similar results have been reported in tumour cell lines originating from different tissue types (Georg *et al.*, 1989). The concentration range of TNF-α used by us and others (Medcalf *et al.*, 1988; Georg *et al.*, 1989) was similar to those found in sera of cancer patients (Balkwill *et al.*, 1987). Of particular interest, we showed that even in cancer cells derived from the same tissue type, i.e. colon carcinoma cells, the pattern of response to cytokine stimulation differed greatly depending on the cell line (Table I and Figure 2). Therefore, hypotheses on the biological role of inflammatory mediators acting on the modulation of cancer cell-associated proteolyis should not be drawn from observations made in one particular cell line or in a limited number of tumour cell types. On the other hand, our findings may explain the contradictory observations which suggest that TNF-α can facilitate or alternatively inhibit tumour progression (Orosz *et al.*, 1993; Quin *et al.*, 1993).

We further characterised the cytokine and cytokine receptor profiles of the eight colon carcinoma cell types (Figure 5). All cell lines expressed the two types of TNF receptor transcripts as evidenced by Northern analysis. Our observation is in agreement with the identification of both TNF receptors in the majority of cell types and tissues (Loescher *et al.*, 1990; Schall *et al.*, 1990; Smith *et al.*, 1990). It is possible, however, that *in vivo*, low levels of TNF-α receptor expression preclude their identification as in the case of breast cancer (Miles *et al.*, 1994). TNF-α, IL-1β and IL-6 transcripts were not detectable, nor were those of IL-1 and IL-6 receptors. To our knowledge, there are only a few reports dealing with cytokine and cytokine receptor expression in colon carcinoma cell lines (Gaffney *et al.*, 1988; Jung *et al.*, 1995). Expression of a large variety of cytokine and cytokine receptor genes was reported in melanoma and sarcoma tumours but using the polymerase chain reaction (Colombo *et al.*, 1992; Pekarek *et al.*, 1993; Mattei *et al.*, 1994). In colon cancer patient tissue samples, TNF-α RNA expression was localised, by *in situ* hybridisation, in tumour-infiltrating stromal macrophages (Beissert *et al.*, 1989; Naylor *et al.*, 1990). No TNF-α transcript was detected in the eight human colon carcinoma cell lines and using a cytotoxic/cytostatic assay involving TNF-susceptible WEHI 164 fibrosarcoma cells, we found no TNF-α-dependent activity in 10-fold concentrated conditioned media (data not shown). All these cell lines responded to TNF-α by increasing the expression of proteins implicated in the plasminogen activation.

We observed that colon carcinoma cell lines which responded to IL-1β, did not exhibit detectable levels of its receptors. There are two known receptors for IL-1, the type 1 is believed to mediate the biological responses, whereas the function of the type 2 receptor is still unclear (Sims *et al.*, 1989; Deyerle *et al.*, 1992; Sims *et al.*, 1993). Many IL-1-sensitive cells are known to express very low levels of type 1 IL-1-R (Sims *et al.*, 1993). Despite low level expression or even undetectable expression of type 1 receptor, these cells

**Figure 3** Dose–response curve of the effect of TNF-$\alpha$ concentration on PA and PAI-1 production in SW 620 and SW 1116 cells. The colon carcinoma cells were plated at $2 \times 10^6$ cells per well and left overnight in FCS-containing media. After washing with serum-free medium, cells were incubated with different concentrations of TNF-$\alpha$. The 24h conditioned media were collected for uPA and PAI-1 EIA. The results, expressed in ng ml$^{-1}$ per $10^6$ cells, are the means of two experiments performed in duplicate. The inserts illustrate the SDS-PAGE zymography of representative conditioned media of cells treated with corresponding TNF-$\alpha$ concentrations. The first two lanes are standards of high and low molecular weight uPA and of tPA.

were capable of responding to IL-1$\beta$ (Deyerle *et al.*, 1992; Sims *et al.*, 1993). Therefore, the absence of IL-1-R mRNA expression in colon carcinoma cells is not contradictory to the IL-1$\beta$-induced enhancement of the plasminogen activation system in these cells.

Based on our observations, we suggest that the macrophage-derived cytokines, TNF-$\alpha$ and IL-1$\beta$, can stimulate the plasminogen activation system of colon carcinoma cells. However, the pattern of response varied greatly depending on the cell type and we could not correlate this with the abundance of one or the other TNF-R type. Moreover, we have not analysed the TNF-R expression upon TNF-$\alpha$ treatment. Internalisation of TNF-R (Imamura *et al.*, 1987), as well as protease-mediated shedding of TNF-R (Ding and Porteu, 1992), followed by resynthesis of newly formed receptors by monocytes under TNF-$\alpha$ stimulation have been described. How such putative regulation may affect the plasminogen activation system expression in colorectal carcinoma cells is not known. The TNF-R55-mediated signalling pathway has been elucidated but the role of TNF-R75 is still under debate (Heller and Krönke, 1994). How the signal mediated by these two receptors modulates the transcriptional activity of the plasminogen activation system also remains unclear. It is suggested that TNF-R75

**Figure 4** Effect of anti-TNF-$\alpha$ Fab fragments on the increased production of uPA in SW 1116 cells. Approximately $4 \times 10^6$ cells per well were plated and left overnight in their FCS-containing media. After washing with serum-free media, cells were incubated for 24 h in the absence (control) or in the presence of TNF-$\alpha$ (10 ng ml$^{-1}$ (+ TNF-$\alpha$) alone or together with anti-TNF-$\alpha$ Fab fragments (10 $\mu$g ml$^{-1}$) (+ anti-TNF-$\alpha$) or non-immune Fab fragments (+ control Fab). The uPA (■) and PAI-1 (□) antigens were determined in the 24 h conditioned media by EIAs. The results are the means ± s.d. of two experiments performed in duplicate.

**Figure 5** Northern analysis of cytokine and cytokine receptor expression in colon carcinoma cells. The polyA$^+$ RNA extracted from colon carcinoma cell lines cultured in FCS-containing media was analysed by Northern blot hybridisation using probes for TNF-$\alpha$, IL-1$\beta$, IL-6, TNF-$\alpha$ receptors 1 and 2 (TNF-$R_1$ and -$R_2$), IL-1 receptors 1 and 2, IL-6 receptor and $\beta$-actin as described in the Materials and methods section. Only autoradiograms of the TNF receptor hybridisation are illustrated. No signal was obtained with the other probes even after 2–3 weeks of autoradiography exposure.

might recruit TNF-α for signalling through TNF-R55 (Tartaglia *et al.*, 1993) or alternatively, TNF-R75 might act by its own signalling pathway and by regulating the access of TNF-α to TNF-R55 (Bigda *et al.*, 1994; Erikson *et al.*, 1994).

In view of our results, we hypothesise that activated tumour-infiltrating macrophages secreting TNF-α and IL-1β may modulate positively or negatively the plasminogen activation associated with cancer cells, thus favouring or interfering with the degradation of surrounding peritumoral tissues. The paracrine activation of cancer cell plasminogen-dependent proteolysis may be important in determining the invasive and metastatic phenotype of colon cancer.

**Acknowledgements**
The authors thank Dr V Jongeneel for helpful and critical review of the manuscript; Dr F Lejeune, Dr R McDonald and Dr D Heumann for generous gifts of reagents; L Kolly and D Bachmann for excellent technical assistance and S Cherpillod for typing the manuscript. This work was supported by the Swiss Cancer League (For 49), the 'Ligue Neuchâteloise contre le Cancer', Cancer Research Switzerland (AKT432), the Fondation Emma Muschamp (to HL), the Swiss Science Foundation (31.266.42.89 and 32.290.34.90 to BS and 31-40889.94 to EKOK) and the Charles Veillon Foundation.

### References

BALKWILL F, BURKE F, TALBOT D, TAVERNIER J, OSBORNE R, NAYLOR S, DURBIN H AND FIERS W. (1987). Evidence for tumour necrosis factor/cachectin production in cancer. *Lancet*, **2**, 1229-1232.

BEISSERT S, BERGHOLZ M, WAASE I, LEPSIEN G, SCHAUER A, PFIZENMAIER K, AND KRÖNKE M. (1989). Regulation of tumor necrosis factor gene expression in colon adenocarcinoma: *in vivo* analysis by *in situ* hybridization. *Proc. Natl Acad. Sci. USA*, **86**, 5064-5068.

BIGDA J, BELETSKY I, BRAKEBUSCH C, VVARFOLOMEEV Y, ENGELMANN H, BIGDA J, HOLTMANN H AND WALLACH D. (1994). Dual role of the p75 tumor necrosis factor (TNF) receptor in TNF cytotoxicity. *J. Exp. Med.*, **180**, 445-460.

CAJOT JF, KRUITHOF EKO, SCHLEUNING WD, SORDAT B AND BACHMANN F. (1986). Plasminogen activators, plasminogen activator inhibitors and procoagulant analyzed in twenty human tumor cell lines. *Int. J. Cancer*, **38**, 719-727.

COLOMBO MP, MACCALLI C, MATTEI S, MELANI C, RADRIZZANI M AND PARMIANI G. (1992). Expression of cytokine genes, including IL-6 in human malignant melanoma cell lines. *Melanoma Res.*, **2**, 181-189.

DEYERLE KL, SIMS JE, DOWER SK AND BOTHWELL MA. (1992). Pattern of IL-1 receptor gene expression suggests role in noninflammatory processes. *J. Immunol.*, **149**, 1657-1665.

DING AH AND PORTEU F. (1992). Regulation of tumor necrosis factor receptors on phagocytes. *Proc. Soc. Exp. Biol. Med.*, **200**, 458-465.

DINGEMANS KP, ZEEMAN-BOESCHOTTEN IM, KEEP RF AND DAS PK. (1993). Transplantation of colon carcinoma into granulation tissue induces an invasive morphotype. *Int. J. Cancer*, **54**, 1010-1016.

ERICKSON SL, DE SAUVAGE FJ, KIKLY K, CARVER-MOORE K, PITTS-MEEK S, GILLETT N, SHEEHAN KCF, SCHREIBER RD, GOEDDEL DV AND MOORE MW. (1994). Decreased sensitivity to tumour-necrosis factor but normal T-cell development in TNF receptor-2-deficient mice. *Nature*, **372**, 560-563.

GAFFNEY EV, KOCH G, TSAI S-C, LOUCKS T AND LINGENFELTER SE. (1988). Correlation between human cell growth response to interleukin 1 and receptor binding. *Cancer Res.*, **48**, 5455-5459.

GANESH S, SIER CFM, GERRIT GRIFFIOEN JM, VLOEDGRAVEN HJM, DE BOER A, WELVAART K, VAN DE VELDE CJH, VAN KRIEKEN JHJM, LAMERS CBHW AND VERSPAGET HW. (1994). Prognostic relevance of plasminogen activators and their inhibitors in colon cancer. *Cancer Res.*, **54**, 4065-4071.

GEORG B, HELSETH E, LUND LR, SKANDSEN T, RICCIO A, DANO K, UNSGAARD G AND ANDREASEN PA. (1989). Tumor necrosis factor-α regulates mRNA for urokinase-type plasminogen activator and type-1 plasminogen activator inhibitor in human neoplastic cell lines. *Mol. Cell. Endocrinol.*, **61**, 87-96.

HASDAY JD, SHAH EM AND LIEBERMAN AP. (1990). Macrophage tumor necrosis factor-α release is induced by contact with some tumors. *J. Immunol.*, **145**, 371-379.

HEALY AM AND GELEHRTER TD. (1994). Induction of plasminogen activator inhibitor-1 in HepG2 human hepatoma cells by mediators of the acute phase response. *J. Biol. Chem.*, **269**, 19095-19100.

HEINRICH PC, CASTELL JV AND ANDUS T. (1990). Interleukin-6 and the acute phase response. *Biochem. J.*, **265**, 621-636.

HELLER RA AND KRÖNKE M. (1994). Tumor necrosis factor-mediated signaling pathways. *J. Cell Biol.*, **126**, 5-9.

HEUSSEN C AND DOWDLE EB. (1980). Electrophoretic analysis of plasminogen activators in polyacrylamide gels containing sodium dodecyl sulfate and copolymerized substrates. *Anal. Biochem.*, **102**, 196-202.

IMAMURA K, SPRIGGS D AND KUFE D. (1987). Expression of tumor necrosis factor receptors on human monocytes and internalization of receptor bound ligand. *J. Immunol.*, **139**, 2989-2992.

JUNG HC, ECKMANN L, YANG SK, PANJA A, FIERER J, MORZYCKA-WROBLEWSKA E AND KAGNOFF MF. (1995). A distinct array of proinflammatory cytokines is expressed in human colon epithelial cells in response to bacterial invasion. *J. Clin. Invest.*, **95**, 55-65.

LEVINE S AND SALTZMAN A. (1990). Lymphatic metastases from the peritoneal cavity are increased in the postinflammatory state. *Inv. Metast.*, **10**, 281-288.

LOETSCHER H, PAN YCE, LAHM HW, GENTZ R, BROCKHAUS M, TABUCHI H AND LESSLAUER W. (1990). Molecular cloning and expression of the human 55 kd tumor necrosis factor receptor. *Cell*, **61**, 351-359.

LOSKUTOFF DJ. (1991). Regulation of PAI-1 gene expression. *Fibrinolysis*, **5**, 197-206.

MANTOVANI A, BOTTAZZI B, COLOTTA F, SOZZANI S AND RUCO L. (1992). The origin and function of tumor-associated macrophages. *Immunol. Today*, **13**, 265-270.

MARSHALL, BC, XU QP, RAO NV, BROWN BR AND HOIDAL JR. (1992). Pulmonary epithelial cell urokinase-type plasminogen activator induction by interleukin-1β and tumor necrosis factor-α. *J. Biol. Chem.*, **267**, 11462-11469.

MASURE S AND OPDENAKKER G. (1989). Cytokine-mediated proteolysis in tissue remodelling. *Experientia*, **45**, 542-549.

MATTEI S, COLOMBO MP, MELANI C, SILVANI A, PARMIANI G AND HERLYN M. (1994). Expression of cytokine/growth factors and their receptors in human melanoma and melanocytes. *Int. J. Cancer*, **56**, 853-857.

MCMAHAN CJ, SLACK JL, MOSLEY B, COSMAN D, LUPTON SD, BRUNTON LL, GRUBIN CE, WIGNALL JL, JENKINS NA, BRANNAN CI, COPELAND NG, HUEBNER K, CROCE CM, CANNIZZARRO LA, BENJAMIN D, DOWER SK, SPRIGGS MK AND SIMS JE. (1991). A novel IL-1 receptor, cloned from B cells by mammalian expression is expressed in many cell types. *EMBO J.*, **10**, 2821-2832.

MEDCALF RL, KRUITHOF EKO AND SCHLEUNING WD. (1988). Plasminogen activator inhibitor 1 and 2 are tumor necrosis factor/cachectin-responsive genes. *J. Exp. Med.*, **168**, 751-759.

MILES DW, HAPPERFIELD LC, NAYLOR MS, BORROW LG, RUBENS RD AND BALKWILL FR. (1994). Expression of tumour necrosis factor (TNFα) and its receptors in benign and malignant breast tissue. *Int. J. Cancer*, **56**, 777-782.

NAKAZAKI H. (1991). Preoperative and postoperative cytokines in patients with cancer. *Cancer*, **70**, 709-713.

NAYLOR MS, STAMP GWH AND BALKWILL FR. (1990). Investigation of cytokine gene expression in human colon cancer. *Cancer Res.*, **50**, 4436-4440.

NAYLOR MS, STAMP GW, FOULKES WD, ECCLES D AND BALKWILL FR. (1993). Tumour necrosis factor and its receptors in human ovarian cancer: potential role in disease progression. *J. Clin. Invest.*, **91**, 2194-2206.

NIEDBALA MJ AND PICARELLA MS. (1992). Tumour necrosis factor induction of endothelial cell urokinase-type plasminogen activator mediated proteolysis of extracellular matrix and its antagonism by γ-interferon. *Blood*, **79**, 678-687.

OPDENAKKER G AND DAMNE JV. (1992). Cytokines and proteases in invasive processes: Molecular similarities between inflammation and cancer. *Cytokine*, **4**, 251-258.

OROSZ P, ECHTENACHER B, FALK W, RÜSCHOFF J, WEBER D AND MÄNNEL DN. (1993). Enhancement of experimental metastasis by tumor necrosis factor. *J. Exp. Med.*, **177**, 1391-1398.

PEKAREK LA, WEICHSELBAUM RR, BECKETT MA, NACHMAN J AND SCHREIBER H. (1993). Footprinting of individual tumors and their variants by constitutive cytokine expression patterns. *Cancer Res.*, **53**, 1978-1981.

PERLMUTTER DH, DINARELLO CA, PUNSAL PI AND COLTEN HR. (1986). Cachectin/tumor necrosis factor regulates hepatic acute-phase gene expression. *J. Clin. Invest.*, **78**, 1349-1354.

QUIN Z, KRÜGER-KRASAGAKES S, KUNZENDORF U, HOCK H, DIAMANTSTEIN T AND BLANKENSTEIN T. (1993). Expression of tumor necrosis factor by different tumor cell lines results either in tumor suppression or augmented metastasis. *J. Exp. Med.*, **178**, 355-360.

SCHALL TJ, LEWIS MM, KOLLER KJ, LEE A, RICE GC, WONG GHW, GATANAGA T, GRANGER GA, LENTZ R, RAAB H, KOHR WJ AND GOEDDEL D. (1990). Molecular cloning and expression of a receptor for human tumor necrosis factor. *Cell*, **61**, 361-370.

SCHLEEF RR, BEVILACQUA MP, SAWDEY M, GIMBRONE JR MA AND LOSKUTOFF DJ. (1988). Cytokine activation of vascular endothelium. Effects on tissue-type plasminogen activator and type 1 plasminogen activator inhibitor. *J. Biol. Chem.*, **263**, 5797-5803.

SIMS JE, MARCH KJ, COSMAN D, WIDMER MB, MACDONALD HR, MCMAHAN CJ, GRUBIN CE, WIGNALL JM, JACKSON JL, CALL SM, FRIEND D, ALPERT AR, GILLIS S, URDAL DL AND DOWER SK. (1988). cDNA expression cloning of the IL-1 receptor, a member of the immunoglobulin superfamily. *Science*, **241**, 585-589.

SIMS JE, ACRES B, GRUBIN CE, MCMAHAN CJ, WIGNALL JM, MARCH CJ AND DOWER SK. (1989). Cloning the interleukin 1 receptor from human T cells. *Proc. Natl Acad. Sci. USA*, **86**, 8946-8950.

SIMS JE, GAYLE MA, SLACK JL, ALDERSON MR, BIRD TA, GIRI JG, COLOTTA F, RE F, MANTOVANI A, SHANEBECK K, GRABSTEIN KH AND DOWER SK. (1993). Interleukin 1 signaling occurs exclusively via the type 1 receptor. *Proc. Natl Acad. Sci. USA*, **90**, 6155-6159.

SITRIN RG, TOD III RF, MIZUKAMI IF, GROSS TJ, SCHOLLENBERGER SB AND GYETKO MR. (1994). Cytokine-specific regulation of urokinase receptor (CD 87) expression by U937 mononuclear phagocytes. *Blood*, **84**, 1268-1275.

SMITH CA, DAVIS T, ANDERSON D, SOLAM L, BECKMAN PM, JERZY R, DOWER SK, COSMAN D AND GOODWIN RG. (1990). A receptor for TNF defines an unusual family of cellular and viral proteins. *Science*, **248**, 1019-1023.

TADA M, DISERENS AC, DESBAILLETS I AND DE TRIBOLET N. (1994). Analysis of cytokine receptor mRNA expressions in human glioblastoma cells and normal astrocytes by reverse transcription polymerase chain reaction. *J. Neurosurg.*, **80**, 1063-1073.

TARTAGLIA LA, PENNICA D AND GOEDDEL DV. (1993). Ligand passing: the 75-kDa tumor necrosis factor (TNF) receptor recruits TNF for signaling by the 55-kDa TNF receptor. *J. Biol. Chem.*, **268**, 18542-18548.

TRAN-THANG C, VOUILLAMOZ D, KRUITHOF EKO AND SORDAT B. (1994). Degradation of laminin by human colon carcinoma cells mediated by tissue-type plasminogen activator is cell-associated. *J. Cell. Physiol.*, **161**, 285-292.

VAN DER BOSCH J, RÜLLER E, ERNST M, SCHADE UF, MATHISON JC, RÜLLER S AND SCHLAAK M. (1992). Cytokines involved in monocyte mediated tumor cell death and growth inhibition in serum-free medium. *J. Cell. Physiol.*, **152**, 617-625.

VAN HINSBERGH VWM, VAN DEN BERG EA, FIERS W AND DOOIJEWAARD G. (1990). Tumor necrosis factor induces the production of urokinase-type plasminogen activator by human endothelial cells. *Blood*, **75**, 1991-1998.

VAN SNICK J, CAYPHAS S, VINK A, UYTTENHOVE C, COULIE PG, RUBIRA MR, AND SIMPSON RJ. (1986). Purification and $NH_2$-terminal amino acid sequence of a T-cell-derived lymphokine with growth factor activity for B-cell hybridomas. *Proc. Natl Acad. Sci. USA*, **83**, 9679-9683.

VASSALLI P. (1992). The pathophysiology of tumor necrosis factor. *Annu. Rev. Immunol.*, **10**, 411-452.

WALTZ DA, SAILOR LZ AND CHAPMAN HA. (1993). Cytokines induce urokinase-dependent adhesion of human myeloid cells. A regulatory role for plasminogen activator inhibitors. *J. Clin. Invest.*, **91**, 1541-1552.

YAMASAKI K, TAGA T, HIRATA Y, YAWATA H, KAWANISHI Y, SEED B, TANIGUCHI T, HIRANO T AND KISHIMOTO T. (1988). Cloning and expression of the human interleukin-6 (BSF-2/IFNβ 2) receptor. *Science*, **241**, 825-828.